# Stochastic Feedback Based Continuous-Discrete Cubature Kalman Filtering for Bearings-Only Tracking

**DOI:** 10.3390/s18061959

**Published:** 2018-06-17

**Authors:** Renke He, Shuxin Chen, Hao Wu, Lei Hong, Kun Chen

**Affiliations:** 1 Information and Navigation College, Air Force Engineering University, Xi’an 710077, China; lnzrds@163.com (R.H.); chenshuxin68@163.com (S.C.); HLei0525@163.com (L.H.); 2 Air Force Harbin Flight Academy, Harbin 150006, China; kunchen365@sina.com

**Keywords:** cubature Kalman filtering, bearings-only tracking, feedback, nonlinear filtering, continuous-discrete systems

## Abstract

Bearings-only tracking only adopts measurements from angle sensors to realize target tracking, thus, the accuracy of the state prediction has a significant influence on the final results of filtering. There exist unpredictable approximation errors in the process of filtering due to state propagation, discretization, linearization or other adverse effects. The idea of online covariance adaption is proposed in this work, where the post covariance information is proved to be effective for the covariance adaption. With theoretical deduction, the relationship between the posterior covariance and the priori covariance is investigated; the priori covariance is modified online based on the feedback rule of covariance updating. The general framework integrates the continuous-discrete cubature Kalman filtering and the feedback rule of covariance updating. Numerical results illustrated that the proposed method has advantages over decreasing unpredictable errors and improving the computational accuracy and efficiency.

## 1. Introduction

Bearings-only tracking (BOT) [1] has universal applications in the field of navigation, especially in passive target tracking. In the BOT process, only angle parameters measured by the angle sensors (such as passive angle sensor, sonar, antenna array, sensor network, infrared sensor) are adopted to achieve the localization and tracking of the target object. The accuracy of state prediction and measurement information are two main factors that influence results of the target tracking. In recent years increasing accuracy of bearings with the development of the passive angle sensors has been witnessed. Generally, filtering methods describe the state process in a discretized form, such as extended Kalman filtering (EKF) [2], unscented Kalman filtering (UKF) [3], particle filter (PF) [4], cubature Kalman filtering (CKF) [5] and so on. The form is propitious for calculation and is convenient to implement mathematical deductions. However, the state process of a BOT system is continuous, yet the measurement process is discrete, and discretization of state process would inevitably lead to the increase of estimation errors. Thus, the continuous-discrete (CD) filtering methods are more suitable to solve a BOT problem than those traditional filtering methods. The CD filtering methods can also be applied for target tracking [6], finance [7], stochastic control [8], etc.

While state models for most target tracking algorithms are described as stochastic differential equations (SDEs), CD filtering methods are quite different from traditional methods. Thus, mathematical models for CD filtering methods are more complicated than traditional ones, while the CD methods are potentially more accurate [9]. The common form of the stochastic system can be expressed in the form of stochastic differential equation (SDE):

(1) dx(t)=F(x(t),t)dt+G(t)dw(t),

where $x(t)\in {\mathbb{R}}^{n}$ is the *n*-dimensional target state vector, $F:{\mathbb{R}}^{n}\times {\mathbb{R}}^{p}\to {\mathbb{R}}^{n}$ is known as the drift function, G(t) is the diffusion matrix, and w(t) is a Brownian motion, which is known as a Wiener process. The alternative form is shown below:

(2) $$\frac{dx(t)}{\mathrm{dt}}=F(x(t),t)+G(t)\frac{dw(t)}{\mathrm{dt}}$$

This form known as Langevin form in Physics is widely used, while the derivative of the Wiener term $\frac{dw(t)}{\mathrm{dt}}$ actually does not exist formally. It should be written as Equation (1). Moreover, Equation (1) cannot be solved simply as the following integral function if the diffusion matrix is a random process related to noises.

(3) $$x(t)=x(t_{0})+\int_{t_{0}}^{t}F(x(s),s)\mathrm{ds}+\int_{t_{0}}^{t}G(x(s),s)dw(t)$$

where the covariance (suppose it is 1-dimension) of the Wiener process is related to *t*, the value of the covariance will trend to be infinitely large; therefore, it makes no sense for the second item to be calculated based on the rules of Riemann--Stieltjes calculus. The SDEs problem mentioned above can be solved by Itô calculus and Stratonovich calculus [10], the difference between them can be referred to in Reference [11]. These characteristics distinguish the CD filtering methods from the discrete time domain filtering methods.

Many numerical methods are applied to solve approximately the SDEs problem in continuous time domain filtering methods. Taylor approximation and Runge-Kutta approximation are two main numerical methods which are widely used to design continuous time domain filtering methods. In Reference [12], the order 0.5 Euler-Maruyama method was proposed, the Euler scheme of SDEs was applied in the method to achieve the 0.5 rate of convergence. Similarly, the order 1.5 strong Taylor approximations and CKF are integrated in Reference [13] to propose continuous-discrete CKF (CD-CKF); however, the computation efficiency is compromised by too many derivative operations in the process of state propagating. The deterministic Runge-Kutta methods and a moment matching technique are used for continuous-time cubature Kalman filters in Reference [14], where the non-additive process noise is considered. The accurate and effective implementation of the filtering method is a key to most researches. In Reference [15], an efficient embedded Runge-Kutta pair and automatic global error control are proposed to improve performance of the complex computational procedure. Similarly, the global error control is proposed to enhance the accuracy and the robustness of the continuous-discrete extended Kalman filter (CD-EKF) in Reference [6]. The Explicit Singly Diagonally Implicit Runge-Kutta (ESDIRK) integrator with sensitivity analysis capabilities in Reference [16] was proved to be efficient to solve nonlinear continuous-discrete filtering problems for stochastic systems, in which the internal integration step is chosen by the step-size controller for Runge-Kutta method. The step-size control technology was also adopted in Reference [17], and the variable-step-size Gauss- and Lobatto-type nested implicit Runge-Kutta formulas of orders four and six are built within the EKF framework. Automatic local and global error regulation mechanisms were implemented in Reference [16,17], which are similar to the notion of accurate continuous-discrete extended Kalman filtering (ACD-EKF) proposed by Kulikov and Kulikova [6,15]. There exist the unpredictable errors due to discretization, linearization, several state predictions in measurement intervals or other causes, which are not considered in the above methods. In Reference [18], Kalman fitler (KF) and CKF are integrated to reduce the problem of the amount of calculations, low accuracy and poor convergence in discrete-time domain, and the adaptive fading factor to adjust the error covariance. In Reference [19], the covariance adaption scheme within the EKF framework was presented to solve the problem. However, the details of the state propagation in continuous-discrete framework are not fully considered, and undoubtedly, larger errors occur when EKF is applied to deal with nonlinear problems.

To alleviate the effects of unpredictable errors, the stochastic feedback of continuous discrete cubature Kalman filter (SFCD-CKF) is proposed in this paper. Compared with the CD-CKF and other CD filtering methods, SFCD-CKF adopts posterior information in the stochastic feedback framework to adapt the priori error covariance online, and online adaption decreases the costly computation during the approximation process and improves the accuracy of state estimation. The paper is structured as follows: Section 2 presents the BOT model, Section 3 briefly introduces general types of continuous-discrete filters, and Section 4 deduces covariance updating and presents stochastic feedback framework of CD-CKF. Simulations results are shown in Section 5, and Section 6 concludes the main work.

## 2. The Model of BOT in Continuous-Discrete Form

Suppose that the positions are set in a 2-dimensional Cartesian coordinate, the measurement information is provided by the observer with the passive angel sensor, the state of target is: $x_{}^{t}(t)={\left[x_{}^{t}(t),y_{}^{t}(t),{\dot{x}}^{t}(t),{\dot{y}}^{t}(t)\right]}^{T}$, where the position and velocity are represented by vector: $p_{}^{t}(t)={\left[x_{}^{t}(t),y_{}^{t}(t)\right]}^{T}$ and $v_{}^{t}(t)={\left[{\dot{x}}^{t}(t),{\dot{y}}^{t}(t)\right]}^{T}$ respectively. The state of the observer is: $x_{}^{s}(t)={\left[x_{}^{s}(t),y_{}^{s}(t),{\dot{x}}^{s}(t),{\dot{y}}^{s}(t)\right]}^{T}$, and the related state is: $x(t)=x_{}^{t}(t)-x_{}^{s}(t)={\left[x(t),y(t),\dot{x}(t),\dot{y}(t)\right]}^{T}$.

The state equation of the bearings-only tracking system is given by a stochastic differential equation:

(4) $$dx(t)=f(x(t),t)\mathrm{dt}+\sqrt{Q}dw(t),$$

where Q is the diffusion matrix, the other parameters are the same as Equation (1). The measurements can be described in a discrete time form:

(5) $$z_{k}=h(x_{k},k)+\omega_{k},\;k=1,2,\cdots$$

(6) $$h(x_{k},k)=\arctan\frac{x_{k}}{y_{k}}$$

where the measurement noise $\omega_{k}$ is assumed to follow independent Gaussian distribution with zero mean and known covariance matrix $\sigma^{2}$.

## 3. General Types of Continuous-Discrete Filters

### 3.1. The Taylor Approximation

The Taylor approximation and Runge-Kutta approximation are two main approaches applied to CD filters. The forms of the approximation equations used in these approaches are quite different, and details of Taylor approximation are illustrated below.

The CD-EKF [20] shows the general form of the Taylor approximation. For the time interval (t,t+δ), the state is:

(7) $$x(t+\delta )=x(t)+\delta f(x(t),t)+\sqrt{Q}\beta ,$$

where β is the Gaussian random variable, which is independent of the state. The expectation can be described as:

(8) E[x(t+δ)]=E[x(t)]+δE[f(x(t),t)].

Let $t=t_{k}$ and $t+{\delta =t}_{k+1}$, the covariance matrix is:

(9) var[x(t+δ)]=var[x(t)+δf(x(t),t)]+δQ,

where δ=T is the measurement sampling interval. f(x(t),t) is extended around the known estimate ${\hat{x}}_{k|k}=E[x_{k}|z_{1:k}]$, which is:

(10) $$f(x(t),t)=f({\hat{x}}_{k|k},t)+f_{x}(x(t)-{\hat{x}}_{k|k})+R_{n}(x(t)),$$

where $f_{x}(x(t)-{\hat{x}}_{k|k})$ is the Jacobian of f, and $R_{n}(x(t))$ are the high-order terms.

The predicted state estimate is:

(11) $${\hat{x}}_{k+1|k}=E[x_{k+1}|z_{1:k}]\approx {\hat{x}}_{k|k}+Tf({\hat{x}}_{k+1|k},k).$$

Then the predicted covariance is:

(12) $$\begin{array}{cc} P_{k+1|k} & =\mathrm{var}[x_{k+1}|z_{1:k}]\\ & \approx (I+Tf_{x}(k))P_{k|k}{\left(I+Tf_{x}(k)\right)}^{T}+TQ \end{array}.$$

The 1.5 order approximation is more accurate and the equation is more complex than the method shown above, but the basic form and the perception for deduction are similar. The state is:

(13) $$\begin{array}{cc} x(t+\delta )= & x(t)+\delta f(x(t),t)\\ & +\frac{1}{2}\delta^{2}(\Gamma_{0}f(x(t),t)+\sqrt{Q}\beta +(\Gamma f(x(t),t)\gamma \end{array},$$

where (β,γ) are the pair of correlated Gaussian random variables. The noise-free process function is defined as:

(14) $$f_{d}(x(t),t)=x_{k}+\delta f(x(t),t)+\frac{1}{2}\delta^{2}(\Gamma_{0}f(x(t),t),$$

where

$$\Gamma_{0}=\frac{\partial }{\partial t}+\sum_{i=1}^{n}f_{i}\frac{\partial }{\partial x_{i}}+\frac{1}{2}\sum_{j,p,q=1}^{n}{\sqrt{Q}}_{p,j}{\sqrt{Q}}_{q,j}\frac{\partial^{2}}{\partial x_{p}\partial x_{q}}$$

$$\Gamma_{j}=\sum_{i=1}^{n}{\sqrt{Q}}_{i,j}\frac{\partial }{\partial x_{i}}.$$

### 3.2. CD-CKF

The cubature rule used in CKF has the advantage in solving nonlinear filtering problems, in which the linearization is not needed in filtering. The 1.5 order Itô-type approximation CD-CKF is easily understood based on the details shown in the last section. For the interval j∈(k,k+1), the predicted state estimate is:

(15) $$\begin{array}{cc} {\hat{x}}_{k|k}^{1} & =E[x_{k}^{1}|z_{1:k}]\\ & \approx E[f_{d}(x_{k},\mathrm{kT})+\sqrt{Q}\beta +\Gamma f(x_{k},\mathrm{kT})\gamma |z_{1:k}] \end{array}.$$

For interval j, the error covariance matrix will also be propagated after the state propagating.

(16) $$\begin{array}{cc} P_{k|k}^{1}\approx & \int_{{\mathbb{R}}^{n}}f_{d}(x_{k},\mathrm{kT})f_{d}^{T}(x_{k},\mathrm{kT})N(x_{k};{\hat{x}}_{k|k},P_{k|k})dx_{k}\\ & +\frac{\delta^{3}}{3}(\Gamma f({\hat{x}}_{k|k},\mathrm{kT})(\Gamma f{\left({\hat{x}}_{k|k},\mathrm{kT}\right)}^{T}\\ & +\frac{\delta^{2}}{2}[\sqrt{Q}(\Gamma f{\left({\hat{x}}_{k|k},\mathrm{kT}\right)}^{T}+(\Gamma f({\hat{x}}_{k|k},\mathrm{kT}){\sqrt{Q}}^{T}]\\ & -({\hat{x}}^{1}{}_{k|k}){\left({\hat{x}}^{1}{}_{k|k}\right)}^{T}+\delta Q \end{array}$$

Based on the cubature rule, the predicted state estimate can be expressed as:

(17) $${\hat{x}}_{k|k}^{1}\approx \frac{1}{2n}\sum_{i=1}^{2n}X_{i,k|k}^{*(1)},$$

(18) $$X_{i,k|k}^{*(1)}{=f}_{d}({\hat{x}}^{}{}_{k|k}+\sqrt{P_{k|k}}\xi_{i},\mathrm{kT}).$$

Then, the predicted state error covariance can be expressed as:

(19) $$\begin{array}{cc} P_{k|k}^{1}\approx & (\Delta X_{k|k}^{*(1)}){\left(\Delta X_{k|k}^{*(1)}\right)}^{T}\\ & +\frac{\delta^{3}}{3}(\Gamma f({\hat{x}}_{k|k},\mathrm{kT})(\Gamma f{\left({\hat{x}}_{k|k},\mathrm{kT}\right)}^{T}\\ & +\frac{\delta^{2}}{2}[(\Gamma f({\hat{x}}_{k|k},\mathrm{kT})Q^{T}+Q(\Gamma f{\left({\hat{x}}_{k|k},\mathrm{kT}\right)}^{T}]+\delta Q \end{array},$$

where

(20) $$\Delta X_{k|k}^{*(1)}=\frac{1}{\sqrt{2n}}\left[\Delta X_{1,k|k}^{*(1)}-{\hat{x}}_{k|k}^{1},\cdots ,\Delta X_{2n,k|k}^{*(1)}-{\hat{x}}_{k|k}^{1}\right].$$

The details of CD-CKF algorithm can be referred to Reference [13].

## 4. Stochastic Feedback Framework of CD-CKF

Although the accuracy of the CD-CKF algorithm is superior over CD-EKF, the unpredictable error is inevitable. In this work, the stochastic feedback framework of CD-CKF is proposed, and the key is that the posteriori information is utilized to adapt the priori error covariance online.

### 4.1. Covariance Adaption

First, the states, the gain, and the covariance at each measurement interval are considered.

Estimate the updated state is:

(21) $${\hat{x}}^{}{}_{k+1|k+1}={\hat{x}}^{}{}_{k+1|k}+W_{k+1}e_{k+1}.$$

The continuous-discrete cubature gain is:

(22) $$W_{k+1}=P_{\mathrm{xz},k+1|k}P_{\mathrm{zz},k+1|k}^{-1},$$

where $P_{\mathrm{xz},k+1|k}$ and $P_{\mathrm{zz},k+1|k}^{}$ are cross-covariance matrix and innovations covariance matrix respectively.

The error covariance matrix is:

(23) $$P_{k+1|k+1}=P_{k+1|k}-W_{k+1}P_{\mathrm{zz},k+1|k}W_{k+1}^{T}.$$

The innovation is:

(24) $$e_{k+1}=z_{k+1}-{\hat{z}}_{k+1|k}.$$

The predicted measurement is:

(25) $${\hat{z}}_{k+1|k}=\frac{1}{2n}\sum_{i=1}^{2n}h(X_{i,k+1|k}^{},k+1)\approx H{\hat{x}}^{}{}_{k+1|k},$$

where $X_{i,k+1|k}^{}$ is the cubature points, and H is the Jacobian matrix of partial derivatives of *h*. The innovations covariance matrix is:

(26) $$P_{\mathrm{zz},k+1|k}^{}=Z_{k+1|k}Z_{k+1|k}^{T}+R_{k+1},$$

(27) $$Z_{k+1|k}=\frac{1}{\sqrt{2n}}\left[Z_{1,k+1|k}^{}-{\hat{z}}_{k+1|k}^{},\cdots ,Z_{2n,k+1|k}^{}-{\hat{z}}_{k+1|k}^{}\right].$$

Substituting Equations (24) and (25) into Equation (26), the innovations covariance matrix can be modified as:

(28) $$P_{\mathrm{zz},k+1|k}^{}\approx HP_{k+1|k}^{}H^{T}+R_{k+1},$$

where $P_{k+1|k}^{}$ is $P_{k|k}^{j}$ when j→k+1.

The cross-covariance matrix is:

(29) $$\begin{array}{cc} P_{\mathrm{xz},k+1|k}^{} & =X_{k+1|k}Z_{k+1|k}^{T}\\ & \frac{1}{\sqrt{2n}}\left[\Delta X_{1,k|k}^{*(k+1)}-{\hat{x}}_{k|k}^{\left(k+1\right)},\cdots ,\Delta X_{2n,k|k}^{*(k+1)}-{\hat{x}}_{k|k}^{\left(k+1\right)}\right]\cdot \\ & \frac{1}{\sqrt{2n}}{\left[Z_{1,k+1|k}^{}-{\hat{z}}_{k+1|k}^{},\cdots ,Z_{2n,k+1|k}^{}-{\hat{z}}_{k+1|k}^{}\right]}^{T} \end{array}.$$

Substituting Equation (25) into Equation (29) yields:

(30) $$P_{\mathrm{xz},k+1|k}^{}\approx P_{k|k}^{j}H^{T},\;j=k+1.$$

Namely, $P_{\mathrm{xz},k+1|k}^{}\approx P_{k+1|k}^{}H^{T}$. Then, the continuous-discrete cubature gain is:

(31) $$W_{k+1}\approx P_{k+1|k}^{}H^{T}P_{\mathrm{zz},k+1|k}^{-1}.$$

Secondly, the covariance updated online is considered.

Here, an assumption is presented at first: The covariance $P_{j+1|j}^{}$ is nearly a constant. Actually, the assumption is not stringently valid within the whole filtering process, but it is a perfect simplification method that ensures the explicit and real time online estimation of the covariance. The method was also applied in References [21,22].

Let’s consider a maximum likelihood estimation problem.

(32) $$\begin{array}{cc} L(P_{k+1|k}^{}) & =\ln\;p(e_{k-N+1},\cdots ,e_{k}|P_{k+1|k}^{})\\ & =\sum_{j=k-N+1}^{k}\ln\;p(e_{j}|P_{k+1|k}^{}) \end{array},$$

where N is the time window, $e_{j}$ can be regarded as a normally distributed random variable, so p(⋅) can be written as:

(33) $$p(e_{j}|P_{k+1|k}^{})=\frac{1}{\sqrt{{\left(2\pi \right)}^{m}\left|P_{\mathrm{zz},j+1|j}^{}\right|}}\exp\left(-\frac{1}{2}e_{j}^{T}P_{\mathrm{zz},j+1|j}^{}e_{j}\right).$$

$L(P_{k+1|k}^{})$ is a scalar and $P_{k+1|k}^{}$ is a symmetric with size n×n. For derivative $\Omega_{k+1}=\partial L(P_{k+1|k}^{})/\partial P_{k+1|k}^{}$, the *s*-th row and *t*-th column element of the derivative is:

(34) $$\Omega_{k+1}^{s,t}=-\frac{1}{2}\mathrm{tr}\left\{\sum_{j=k-N+1}^{k}\left[\Theta \cdot \Psi \right]\right\},$$

(35) $$\Theta =P_{\mathrm{zz},j+1|j}^{-1}-P_{\mathrm{zz},j+1|j}^{-1}e_{j}e_{j}^{T}P_{\mathrm{zz},j+1|j}^{-1},$$

(36) $$\Psi =\frac{\partial P_{\mathrm{zz},j+1|j}^{}}{\partial P_{k+1|k}^{s,t}},$$

where $P_{k+1|k}^{s,t}$ is the *s*-th row and *t*-th column element of $P_{k+1|k}^{}$. Then ${\hat{P}}_{k+1|k}^{}$ can be yielded by setting $\Omega_{k+1}$ to zero, i.e., $\Omega_{k+1}^{s,t}=0$.

Because $R_{k+1}$ and *H* are independent of $P_{k+1|k}^{}$, with Equations (28) and (36),

(37) $$\Psi =H\frac{\partial P_{k+1|k}^{}}{\partial P_{k+1|k}^{s,t}}H^{T},$$

(38) $$\mathrm{tr}\left\{\sum_{j=k-N+1}^{k}\left[\Theta \cdot H\frac{\partial P_{k+1|k}^{}}{\partial P_{k+1|k}^{s,t}}H^{T}\right]\right\}=0.$$

Then pre- and post-multiply the matrix inside tr{·} by $H^{T}$ and its inverse (or the generalized inverse) can be expressed as:

(39) $$\mathrm{tr}\left\{\sum_{j=k-N+1}^{k}\left[H^{T}\Theta \cdot H\frac{\partial P_{k+1|k}^{}}{\partial P_{k+1|k}^{s,t}}\right]\right\}=0,$$

because $\frac{\partial P_{k+1|k}^{}}{\partial P_{k+1|k}^{s,t}}$ is a constant matrix and its *s*-th row and *t*-th column element is one while other elements are zero. Based on the multiplication rule, the *t*-th column of the matrix $H^{T}\Theta H\cdot \frac{\partial P_{k+1|k}^{}}{\partial P_{k+1|k}^{s,t}}$ cannot be zero while the other column must be zero. So, the value of tr{·} is equal to the *t*-th diagonal element of {·}:

(40) $${\left\{\sum_{j=k-N+1}^{k}\left[H^{T}\Theta \cdot H\frac{\partial P_{k+1|k}^{}}{\partial P_{k+1|k}^{s,t}}\right]\right\}}^{t,t}=0.$$

Furthermore, because the *s*-th row and *t*-th column element of $\frac{\partial P_{k+1|k}^{}}{\partial P_{k+1|k}^{s,t}}$ is one and other elements are zero to ensure elements of {·} to be zero, we can obtain:

(41) $$\sum_{j=k-N+1}^{k}{\left\{\left[H^{T}\Theta \cdot H\right]\right\}}^{t,s}=0,$$

where *t*, *s* can be any value within (0,*n*), so:

(42) $$\sum_{j=k-N+1}^{k}\left[H^{T}\Theta \cdot H\right]=0.$$

Let us multiply both sides of Equation (42) by $P_{j+1|j}^{}$, we get:

(43) $$\sum_{j=k-N+1}^{k}\left[P_{j+1|j}^{}H^{T}\Theta \cdot HP_{j+1|j}^{}\right]=0.$$

With Equations (31) and (35), we have:

(44) $$\sum_{j=k-N+1}^{k}\left[W_{j+1}HP_{j+1|j}^{}-W_{j+1}e_{j}e_{j}^{T}W_{j+1}^{T}\right]=0.$$

Based on Equations (21) and (23), we can get:

(45) $$W_{j+1}e_{j}=\Delta {\hat{x}}_{j+1}={\hat{x}}^{}{}_{j+1|j+1}-{\hat{x}}^{}{}_{j+1|j},$$

(46) $$\sum_{j=k-N+1}^{k}\left[P_{j+1|j}^{}-P_{j+1|j+1}^{}-\Delta {\hat{x}}_{j+1}\Delta {\hat{x}}_{j+1}^{T}\right]=0.$$

Then:

(47) $$\sum_{j=k-N+1}^{k}P_{j+1|j}^{}=\sum_{j=k-N+1}^{k}\left(P_{j+1|j+1}^{}+\Delta {\hat{x}}_{j+1}\Delta {\hat{x}}_{j+1}^{T}\right).$$

Considering the assumption, $P_{k+1|k}^{}$ can be approximated by:

(48) $${\hat{P}}_{k+1|k}^{}=\frac{1}{N}\sum_{j=k-N+1}^{k}P_{j+1|j}^{}=\sum_{j=k-N+1}^{k}P_{j+1}^{*},$$

where $P_{j+1}^{*}$ is defined as an intermediate matrix. Hence:

(49) $${\hat{P}}_{k+1|k}^{}={\hat{P}}_{k|k-1}^{}+\left(P_{k}^{*}-P_{k-N}^{*}\right).$$

### 4.2. Stochastic Feedback Framework

**Figure 1:**
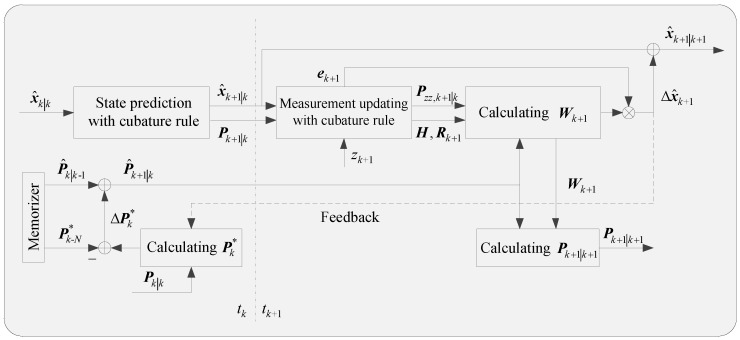
Stochastic feedback framework.

The functions, Equations (47–49), constitute a major part of the covariance adaption framework. As shown in Figure 1, the state prediction and measurement updating are completed within CD-CKF, the difference is the covariance prediction process. The post-covariance information $P_{k-N}^{*}$ in the memorizer is used to update the covariance ${\hat{P}}_{k+1|k}^{}$ online, and it is also used to update the cubature gain $W_{k+1}$. Then the $\Delta {\hat{x}}_{k+1}$ is calculated to generate the state estimation ${\hat{x}}_{k+1|k+1}^{}$, and $\Delta {\hat{x}}_{k+1}$ can be used for covariance generation from feedback channel.

Compared with CD-EKF and CD-CKF, the advantages of the stochastic feedback CD-CKF (SFCD-CKF) framework can be concluded as:

(1) Post covariance information is used to decrease the influence of the unpredictable error within continuous-time domain state prediction process, and as a result, it enhances the accuracy of the filtering.
(2) Online covariance updating can decrease the computational complexity in derivative or matrix operation compared with traditional methods.
(3) The cubature rule improves the performance of the filtering when dealing with non-linear problems, and it also provides a more accurate innovation covariance matrix than CD-EKF and other methods.

## 5. Numerical Simulation

To illustrate the performance of the proposed method, the linear and non-linear continuous time domain models with discrete measurements are considered in this section. Specifically, “linear” and “non-linear” described here refer to the type of state models, because the measurement models in BOT are all non-linear in this study. Itô-1.5 order CD-CKF [13], CD-EKF [20] and continuous-discrete adaptive Kalman filter (CD-AKF, which is also called SFCD-CKF) [19] methods are compared in the simulations. The Monte Carlo simulation is set to be 200 times for each scenario.

### 5.1. Linear Model Tracking

The constant velocity (CV) model is used as the linear state model of BOT. The model can be rewritten as:

(50) $$x_{k}=Fx_{k-1}+v_{k-1},$$

where $v_{k-1}$ is a zero-mean Gaussian noise with covariance matrix Q, F is the state transition matrix. The Gaussian noise distribution can approximately describe the state error in real physical space, and Gaussian noise is convenient to be expressed and deduced mathematically. The measurements function is Equation (5). $q=10^{-5}\;{\mathrm{km}}^{2}{/s}^{3}$ is the intensity of process noise, and the measurement noise is $e_{k}\sim N(0,10^{-2})$. The time window *N* is 5. The observer’s initial state is ${\left[0\;m,0\;m,100\;m/s,50\;m/s\right]}^{T}$. Target’s initial state is ${\left[1\times 10^{5}\;m,1\times 10^{5}\;m,40\;m/s,-190\;m/s\right]}^{T}$.

Q=[Δt330Δt2200Δt330Δt22Δt220Δt00Δt220Δt]·q,

$$F=\left[\begin{array}{cccc} 1 & 0 & \Delta t & 0\\ 0 & 1 & 0 & \Delta t\\ 0 & 0 & 1 & 0\\ 0 & 0 & 0 & 1 \end{array}\right].$$

The root mean square error (RMSE) of position is applied to evaluate the accuracy of BOT.

(51) $$RMSE(k)={\left(\frac{1}{N_{\mathrm{MC}}}\sum_{i=1}^{N_{\mathrm{MC}}}{\left\|x_{k}-{\hat{x}}_{k}^{i}\right\|}_{2}^{2}\right)}^{1/2},$$

where $N_{\mathrm{MC}}$ is the number of Monte Carlo simulations, ${\hat{x}}_{k}^{i}$ is the estimation at time *k* for Monte Carlo simulation *i*, $x_{k}$ is the real state of the target.

**Figure 2:**
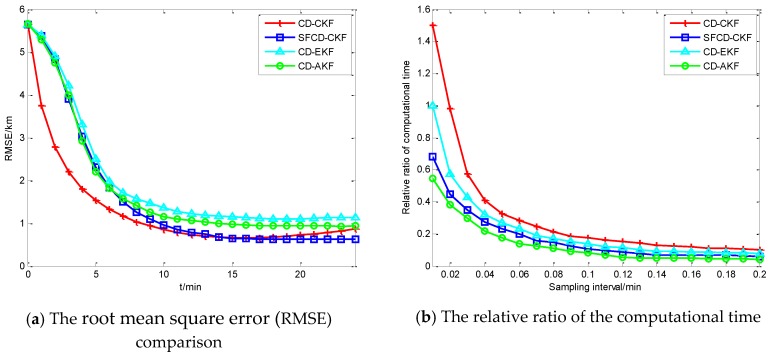
The performance comparison with the CV model.

**Figure 3:**
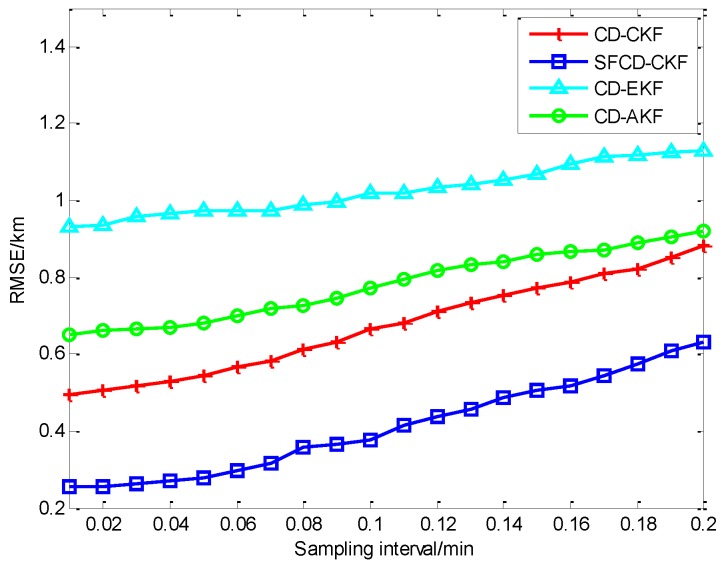
The RMSE with different sampling intervals.

**Figure 4:**
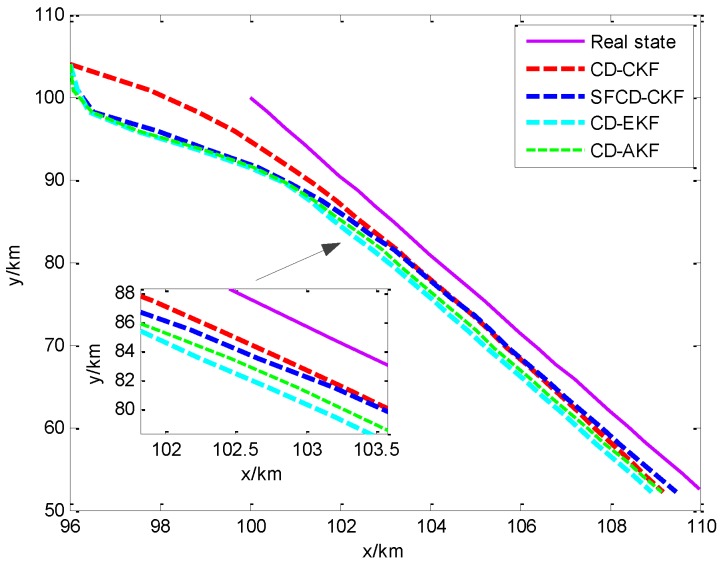
The state estimate with different filtering methods.

As is shown in Figure 2a, the RMSE of CD-CKF decreases faster than the other three methods in the first 10 min. Because of the superior performance of CD-CKF in the nonlinear problem (actually, it is a nonlinear measurement problem in this CV BOT model), CD-CKF is more accurate for filtering in the whole BOT process. The performance of SFCD-CKF is not as good as CD-CKF at the initial moment, while RMSE of SFCD-CKF remains in decline to the end of the process, and the accuracy is higher than the other methods. CD-AKF is a CD-EKF method modified by the idea of covariance online updating, and its accuracy is higher than CD-EKF; however, when compared with CD-CKF and SFCD-CKF, the framework of EKF used in CD filtering is suboptimal, and the accuracy of CD-AKF and CD-EKF are lower comparatively. Figure 3 shows the RMSE with different sampling intervals. In general, the accuracy can be improved with smaller sampling interval, while the computational efficiency would decrease. Figure 4 shows the state estimate with these different filtering methods, where the real state is the track (or path) of the target, and the path length is 48.53 km.

Figure 2b shows the relative ratio of the computational time, and the CD-EKF with sampling interval 0.01 is the datum point. In general, the computational time will decrease with the increase of sampling interval. Because of the complex matrix operation, CD-CKF is the most time-consuming method computationally. CD-AKF and SFCD-CKF exhibit better computational efficiency comparatively, which shows that the framework of covariance online updating has an advantage in improving the computational efficiency.

### 5.2. Nonlinear Model Tracking

In this section, the classical nonlinear model Van der Pol oscillator is used as the state model [15].

(52) $$d\left[\begin{array}{c} x(t)\\ y(t) \end{array}\right]=\left[\begin{array}{c} y(t)\\ \lambda (1-x^{2}(t))y(t)-x(t) \end{array}\right]\mathrm{dt}+\left[\begin{array}{c} 0\\ 1 \end{array}\right]\mathrm{dw}(t),$$

where $y(t)=\dot{x}(t)$ in the Van der Pol oscillator model, and the initial state is ${\left[1,1\right]}^{T}$. The state $x(t)=x(0)+{\left[t,x(t)\right]}^{T}$, where the initial position is $x(0)={\left[1\times 10^{5}\;m,1\times 10^{5}\;m\right]}^{T}$. The parameters are *λ* = 0.3 and $w(t)\sim N(0,10^{-5})$. The measurement is defined by Equation (5).

**Figure 5:**
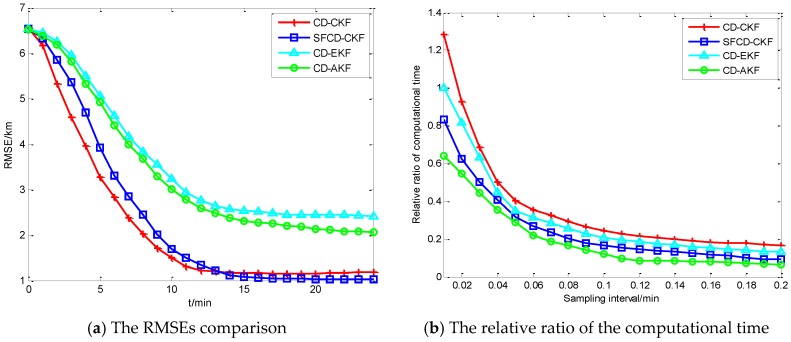
The performance comparison with the nonlinear model.

**Figure 6:**
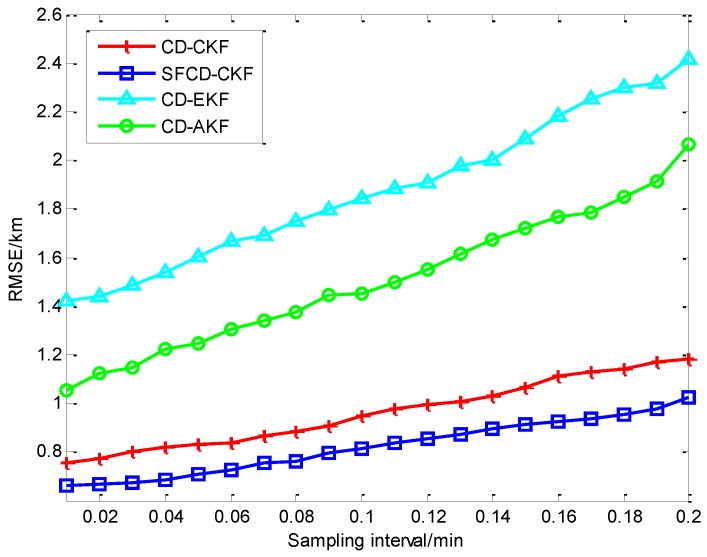
The RMSEs with different sampling intervals.

**Figure 7:**
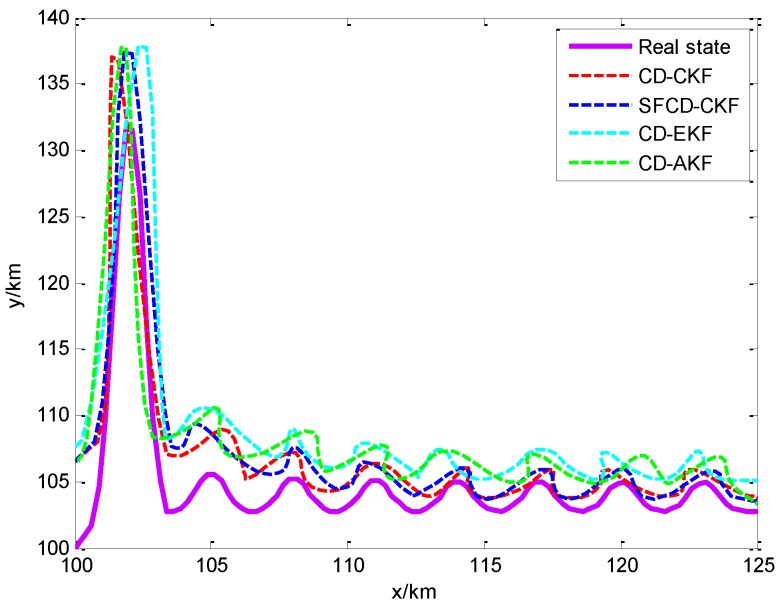
The state estimate with different filtering methods.

Figure 5a shows the different RMSEs of four methods when dealing with a nonlinear filtering problem. CD-CKF and SFCD-CKF show the advantage for nonlinear filtering. In the last stage of the target tracking process, the accuracy of SFCD-CKF is slightly higher than CD-CKF. As we can see from Figure 2a and Figure 5a, the performance of SFCD-CKF is superior over CD-CKF only when the error approaches zero. This indicates that the online covariance updating has an advantage in state estimation with micro variation of the covariance because the approach is sensitive to unpredictable errors in this moment than in the moments when the covariance changes rapidly. The computational efficiency of SFCD-CKF and CD-AKF are demonstrated to be better than the other two methods in Figure 5b. Figure 6 shows the RMSE with different sampling intervals. The accuracy of CD-EKF and CD-AKF are improved rapidly along with the decrease of the sampling intervals, because the error of EKF framework used by CD-EKF and CD-AKF is large when dealing with the nonlinear filtering problem, and the impact of sampling interval on estimation accuracy is significant. Figure 7 shows the state estimate with these different filtering methods, where the real state is the track (or path) of the target. The path length is 133.01 km. With superior computational efficiency, SFCD-CKF generally has an advantage in solving unpredictable error problems.

## 6. Conclusions

Considering the unpredictable error within the process of CD filtering, the framework of online covariance updating is proposed. The key is to use the post information to modify the state estimation. The post covariance information is proved to be valuable for covariance updating by theoretical deduction. The proposed framework integrates CD-CKF with the covariance updating approach, which enhance the performance for the method when implementing the nonlinear filtering, the efficient computation and the correction of unpredictable error. While the approach does not work well when the covariance changes significantly, the adaptive approaches which combine CD-CKF with SFCD-CKF will be the focus of our future research.
